# Withstanding austerity: Equity in health services utilisation in the first stage of the economic recession in Southern Spain

**DOI:** 10.1371/journal.pone.0195293

**Published:** 2018-03-30

**Authors:** Juan Antonio Córdoba-Doña, Antonio Escolar-Pujolar, Miguel San Sebastián, Per E. Gustafsson

**Affiliations:** 1 Delegación Territorial de la Consejería de Salud de la Junta de Andalucía, Cádiz, Spain; 2 Department of Public Health and Clinical Medicine, Epidemiology and Global Health, Umeå University, Umeå, Sweden; University Complutense of Madrid, SPAIN

## Abstract

Scant research is available on the impact of the current economic crisis and austerity policies on inequality in health services utilisation in Europe. This study aimed to describe the trends in horizontal inequity in the use of health services in Andalusia, Spain, during the early years of the Great Recession, and the contribution of demographic, economic and social factors. Consultation with a general practitioner (GP) and specialist, hospitalisation and emergency care were studied through the Andalusian Health Survey 2007 (pre-crisis) and 2011–2012 (crisis), using a composite income index as socioeconomic status (SES) indicator. Horizontal inequity indices (HII) were calculated to take differential healthcare needs into account, and a decomposition analysis of change in inequality between periods was performed. Results showed that before the crisis, the HII was positive (greater access for people with higher SES) for specialist visits but negative (greater access for people with lower SES) in the other three utilisation models. During the crisis no change was observed in inequalities in GP visits, but a pro-poor development was seen for the other types of utilisation, with hospital and emergency care showing significant inequality in favour of low income groups. Overall, the main contributors to pro-poor changes in utilisation were socio-economic variables and poor mental health, due to changes in their elasticities. Our findings show that inequalities in healthcare utilisation largely remained in favour of the less well-off, despite the cuts in welfare benefits and health services provision during the early years of the recession in Andalusia. Further research is needed to monitor the potential impact of such measures in subsequent years.

## Introduction

Along with austerity measures in social investment introduced in many countries, the economic crisis affecting Europe since 2008 has had an impact on many aspects of the mental and physical health of the European population [1]. Likewise, the pressure of the recession and rising healthcare needs across health systems have, in the context of austerity, also had a negative impact in many cases on health service provision [2,3]. Less, however, is known about the effect of the crisis and austerity on social inequalities in healthcare utilisation, an area on which the present study seeks to shed light in the context of Andalusia, Spain.

Several pathways have been proposed to study the effects of global financial crisis on health outcomes, such as income, labour market changes and social welfare conditions. In all of them the introduction of austerity measures by governments may play a crucial role [4]. Recently, Kentikelenis has developed a conceptual model of the multiple ways by which structural adjustment, including austerity measures, has an impact on health and subsequently health inequities [5]. He identified three main pathways: (i) policies directly targeting health systems; (ii) policies with indirect effect on health systems; and (iii) policies impacting the social determinants of health. Ruckert and Labonté describe the visible impacts of austerity-driven welfare reforms since 2008 through two main pathways: social welfare cuts and labour market policies [6]. They highlight that a central pathway that connects austerity to health equity is, thus, the restructuring of health services. They document how austerity has exacerbated health inequities in countries affected by increasing cost of care for drugs or via copayments, such as Italy, or by reducing provision by closing or limiting operating hours of facilities, or by staff layoffs, as occurred in Greece [7].

Spain was hit very hard by the recession, unemployment rising from 8.6% to 25.8% between 2007 and 2012, and the ensuing austerity measures progressively introduced by different governments, with public social expenses substantially reduced during the same period. Health services provision and coverage additionally were affected in 2010, with the limitation of the rate of replacement for vacancies in the public sector to 10%, causing a deeper reduction of public health employees, and especially through the Royal Decree-Law 16/2012, which imposed further budget reductions, introduced new co-payments for drugs, and restricted access to coverage for undocumented migrants [8].

As a decentralized health system, the degree of implementation of such austerity measures has varied among different regions in Spain, with some of them failing to observe centrally enforced austerity measures regarding health coverage. Increasing inter-regional variability, depending on budgetary basis and regional policy decisions, also has been observed since 2008 in the allocation of resources, with, for example, the public health budget per capita ranging between 975€ and 1558€ in 2011 among the different Spanish regions [9]. These restrictions led to a staff reduction by 7% in the Andalusian health system between 2009 and 2013 [10]. This picture is mirrored in patient indicators. For example, increases in official waiting times have been detected for many conditions, especially since 2010 [11], and unmet medical needs in Spain increased from 1.9% to 5.7% between 2007 and 2012, which is in stark contrast to the decreasing trends in the European Union-27 (EU-27) [12].

In addition to these bleak developments of the health systems in Europe and Spain, concerns have been raised about increased social inequalities in health and healthcare use in the wake of the crisis and cuts in social spending. Although a recent publication highlights a reduction in income related self-rated health inequalities in Spain since the economic downturn disproportionately affected the earnings of younger and thus healthier population [13], some evidence suggests that the recession and associated cuts may be increasing social inequalities in health [14]. Even if there is not much information available on the differential impact on various population subgroups, data on several European countries indicate that the more affected groups include the low-educated, people with low income and groups with greater health needs, as well as young couples [15]. Moreover, there is a paucity of literature regarding determinants and structural causes of these health inequalities in Europe [16].

When specifically considering health services utilisation in a broader historical perspective, social gradients in the use of health services given similar needs long have been widely observed in diverse geographical and political settings, even in countries with universal health coverage [17], generally showing greater primary care but less specialist service use among low socioeconomic groups compared to their high socioeconomic status (SES) counterparts [18,19]. In Spain, there is some evidence that social inequalities in access to health services did not change substantially in the decade prior to the onset of the current recession [20]. In a study on 18 OECD countries with data from 2009 Spain is one of the most equitable countries regarding the use of GP but one of the most inequitable regarding the use of specialized doctors [21]. Concerning other health services, a study detected an increase in the probability of use of an emergency department in lower social classes and a non-significant decrease in the probability of hospitalisation in this group compared to higher social classes using data from the Spanish Health Survey [22]. Using the same survey, a recent study revealed a decrease in use of specialist consultations and hospitalisations in lower income population while an increase in emergencies utilisation in this group was detected [23]. Assessing avoidable or unjust inequality in healthcare use in the times around the crisis would therefore provide relevant information on the impact of the Great Recession on inequity in the utilisation of health services and potential underlying mechanisms [24].

Our objective thus was, first, to describe the trends in horizontal inequity in access to health services in Andalusia during the early years of the economic crisis and, second, to attribute the changes in inequality to demographic, economic and social factors.

## Methods

### Setting

This study was carried out in Andalusia, the fourth most populated region in Europe and the most populated in Spain, with about 8.5 million inhabitants. Andalusian economic indicators largely are below the European average. For instance, purchasing power standards per inhabitant in percentage of the EU average were 79% in 2007 and 69% in 2012 [12]. Unemployment for both sexes rose in Andalusia from 12.2% in 2006 to 35.8% in 2012 [25], and poverty rates increased from 29.5% in 2008 to 31.0% in 2012, well above the Spanish poverty rate of 22.2% [26].

The Spanish health system is decentralized so that each of the 17 regions has a high degree of autonomy. Health coverage in Andalusia is provided on a universal basis. Visits to the GP or paediatricians, as well as consultations with specialists, including mental health services, emergency services and hospitalisation, are free of charge at the point of use. Co-payment is required only at ambulatory pharmacies, with co-payment rates ranging from 10% for pensioners to 60% for active workers and full exemptions for some groups as long-term unemployed and disabled patients. The GP is the gatekeeper to access to specialists, who, in turn, are the gatekeepers for non-urgent hospitalisations. All primary healthcare and the majority of emergency services are publicly provided and around 95% of publicly funded hospital based services are public [27].

Since the beginning of the economic recession, the Regional Health Authority budget has decreased from 1168€ per capita in 2008 to 997€ in 2013. In order to illustrate the contextual prerequisites of Andalusia, it might be of interest to present the trend in per capita health services indicators during the first years of the economic recession and consequent cuts. GP consultations per adult decreased about 20% between 2007 and 2012, while hospitalisation and surgical procedures decreased more slowly (-12.4% and -11.7%, respectively). On the other hand, an increase in specialist consultation (2.9%), non-hospital emergency attentions (6.0%) and a very high growth in specialized mental health consultations in adults (32.1%) were observed [28] (see Table 1).

**Table 1:** Selected annual indicators of Andalusian Health Service performance. 2007–2012.

	2007	2008	2009	2010	2011	2012	Change 2007–2012	Percent change
Per capita health budget (€)	1168	1132	1095	1049	1202	997	-171,00	-14,64
GP consultations per capita*	7,69	7,54	7,41	6,50	6,27	6,15	-1,54	-20,07
Paediatrician consultations**	5,29	5,23	5,45	4,93	5,02	4,77	-0,52	-9,76
Specialist consultation	1,25	1,26	1,26	1,23	1,26	1,29	0,04	2,93
Hospitalizations	0,07	0,07	0,06	0,06	0,06	0,06	-0,01	-12,39
Non-hospital emergency attentions	0,69	0,69	0,75	0,78	0,69	0,74	0,04	6,01
Hospital emergency attentions	0,45	0,43	0,43	0,42	0,42	0,39	-0,05	-12,18
Surgical procedures	0,06	0,06	0,06	0,06	0,06	0,06	-0,01	-11,68
Mental health consultations*	0,14	0,14	0,15	0,15	0,17	0,18	0,04	32,09

*over 15 year population

**under 16 year population

Source: Andalusian Health Service. Basic Data.

### Sample

We used a repeated cross-sectional design, with two waves of the Andalusian Health Survey [29]: 2007 for the pre-crisis period and 2011 for the crisis period. This survey is conducted every 4 years, including non-institutionalised adults age 16 and older. It uses a probabilistic multistage cluster and stratified sampling procedure, with a design effect of 1.35 for sample size calculations. There were 6511 people interviewed in 2007 and 6507 in 2011–2012. Field substitution was used during the process to compensate for non-response. In our study, the sample was limited to the population of age 25 and older, when greater stability in employment status or occupation is found, yielding 5011 individuals (2589 men and 2422 women) in 2007 and 5243 individuals (2656 men and 2587 women) in 2011–2012. Missing data were exiguous in the variables included in the analyses.

This study is subject to Spanish legislation on data protection [30]. We accessed data with permission from the Regional Health Authority (Secretaría General de Salud Pública de la Junta de Andalucía), from which the data are available to researchers upon request at epidemiologia.csalud@juntadeandalucia.es. All participants gave their written informed consent to be included in the study. Data were unlinked from any personal identification information during the analyses to ensure anonymity. Additionally, as Spanish law provides, the file containing personal data was registered in the General Data Protection Registry [31].

### Variables

All variables were measured through the questionnaire of the Andalusian Health Survey. Four sets of variables were selected, representing (1) health care utilisation outcomes, (2) socioeconomic status indicator, (3) need variables and (4) non-need variables.

#### Healthcare utilisation outcomes

We selected four variables measuring types of healthcare services utilisation, usually employed in access and utilisation studies in OECD countries: family doctor (GP) consultation in the past two weeks; consultation with specialist in the past two weeks; hospitalisation in the last year; and emergency ward attention in the last year. There was no distinction between public and private health services in the survey questions.

#### Socioeconomic indicator

We discarded the use of the income variable included in the survey as the ranking socioeconomic variable for two reasons: the survey provided only a discrete continuous variable with 9 categories and 33% missing values in our sample; and there are concerns about the low quality of self-reported income data in household surveys in Spain, as evident in previous studies showing global consistency indicators scoring under 50% [32].

To address this limitation we developed a composite index as a proxy for income through principal component analysis, using four assets variables together with subjective financial stress. The asset index approach is at present increasingly used also in middle and high income countries [33]. In our case, a relative measure is suitable for ranking and, moreover, there is evidence that such asset-based indices perform reasonably well as a proxy for living standards and so for assessing health inequalities [34].

The assets variables were (i) home ownership (yes/no), (ii) number of cars in the household, with four categories (none, one, two, three or more), (iii) number of bedrooms in the house, and (iv) air-conditioning at home (yes/no). Subjective financial stress was measured in six categories by the response to the question: “Thinking of your household's total income, is your household able to make ends meet, namely, to pay for its usual necessary expenses: (1) with great difficulty, (2) with difficulty, (3) with some difficulty, (4) fairly easily, (5) easily, or (6) very easily?” Only one factor was extracted in the principal component analysis, which accounted for 33.3% of the total variation in the data. Sampling adequacy was tested with the Kaiser-Meyer-Olkin measure (KMO = 0.658) and Bartlett’s test of sphericity was performed (p <.001). The scoring factors of the variables were all in the expected direction. To further assess the validity of the scoring factor we aggregated the composite income index into quintiles and estimated the proportionate distribution of the different assets or characteristics by quintile of the index. Participants in quintile 5 were more likely to have a greater number of assets and greater easiness to make ends meet. We further checked the validity of the index to be used as a SES ranking variable measuring the correlations of the index with the variable income (with 9 categories) available in 6528 participants. Respondents with higher income consistently showed higher values in the index using the overall sample (overall Spearman correlation coefficient = 0.51). We also stratified by sex and size of the municipality to assess the consistency of the association across sexes and rural/urban settings, obtaining similar results (data not shown). Altogether, these results suggested that the composite income index could be used as a valid SES ranking measure for the purposes of our study. The index contained 359 unique values and was later used in the models as a continuous variable.

#### Variables representing healthcare needs

We selected need factors such as demographic characteristics and health measures associated with health care utilisation widely used in epidemiological and health services research [35,36]. The variables of health needs were: sex (1 = women; 0 = men); age was recoded in three groups (young = 25–44 years, middle = 45–64 years, and older = 65 and older) using middle age as the reference category; self-rated health, originally in five categories, was recoded into two categories, 0 = good (very good, good or fair) and 1 = poor (bad or very bad) self-rated health; self-assessed mental health according to standardized mental component score of the SF–12 [37] as a dichotomous variable, categorising the quintile with lower score as poor mental health (= 1) and the rest as good mental health (= 0), with a cut-off point of 47.3 (there is no universally accepted cut-off level, ours is intermediate between the 45 points used for screening of depressive disorder and the 50 points employed for any common mental disorder [38]); presence of chronic diseases (none, one, two or three, and four or more); and having suffered an accident the previous year (1 = yes, 0 = no).

#### Non-need variables

Non-need variables were: educational level (with five categories: no studies—reference category–, up to 5 years, from 6 to 8 years, secondary studies, and university studies), employment status (working—reference category–, unemployed, retired, housework and others); having private health insurance (1 = yes, 0 = no); size of the municipality of residence (less than 10,000 inhabitants—reference category–, between 10,000 and 50,000; more than 50,000 and province capital); and province of residence. Education and working status are both related to physical and psychosocial health [39,40] and have also been found to be related to healthcare use [41,42]. The size of the municipality and the province of residence may influence accessibility to, and consequently utilisation of health services.

### Analysis

Initially we compared the distribution of the independent variables by the four dependent variables in each period, separately for men and women. Chi-square tests were performed.

Using the proxy variable for income as socioeconomic ordering variable, we first calculated a concentration index (*C*) for each outcome variable. The *C* is related to the concentration curve which plots the cumulative percentage of the healthcare utilisation variable on the y-axis and the cumulative percentage of the sample ranked by the socioeconomic variable beginning from the lowest SES on the x-axis. The *C* is computed as twice the area between the curve and the line of equality (the 45° line). The concentration index of actual healthcare use, therefore, was calculated by the following formula [43]

$$C=\frac{2}{n\cdot \mu }\cdot \sum_{i=1}^{n}h_{i}\cdot R_{i}-1=\frac{2}{\mu }\cdot cov\left(y_{i}\cdot R_{i}\right)$$ (1)

where *y*_*i*_ is the measure of healthcare utilization of *i*th individual, *n* the sample size, *μ* the mean healthcare use and *R*_*i*_ the relative fractional rank in the proxy for income of the *i*th individual. The *C* takes a value of 0 if healthcare utilisation distribution is equal. It has a negative value if the concentration curve lies above the line of equality, which indicates a greater concentration of the health utilisation variable among the lower SES group. On the contrary, it takes a positive value if the concentration curve lies below the line of equality, which indicates a greater concentration of the healthcare utilisation among the higher SES group [43].

When using binary health care utilisation outcomes, the bounds of the CI depend on the minimum, the maximum and the mean of the health care variable. To take account of this limitation, we used the corrected version of the CI proposed by Wagstaff, consisting in dividing the CI by 1–*μ*, being *μ* the average of the health care utilisation variable [44].

In a second step, we performed the decomposition of *C* [45]. Furthermore, as O`Donnell et al. indicate [43], with the decomposition approach, the estimation of horizontal inequity and the explanation of inequity can be done simultaneously. As our healthcare use variables are dichotomous, a nonlinear model must be used. Following van Doorslaer et al. [46] and O’Donnell et al. [43], the healthcare utilisation variable (*y*) is predicted through a probit regression model

$$y_{i}=\Phi \left(\alpha +\sum_{}\beta_{j}^{}x_{ji}+\sum_{k}\gamma_{k}z_{ki}\right)+u_{i}$$ (2)

where Φ is the cumulative normal distribution function, *x* represents need variables and *z* control variables. When a nonlinear model is used, as is our case, the decomposition is possible only by means of a linear approximation, such as the estimation of the partial effects evaluated at the sample means [46]. The linear approximation of Eq (2) is

$$y_{i}=\alpha^{m}+\sum_{j}\beta_{j}^{m}x_{ji}+\sum_{k}\gamma_{k}^{m}z_{ki}+u_{i}$$ (3)

where $\beta_{j}^{m}$ and $\gamma_{k}^{m}$ are the partial effects of each variable treated as fixed parameters and evaluated at the mean, and *u*_*i*_ is the error term. Because Eq (3) is linearly additive, the decomposition can be applied and the CI for *y* can be written as

$$C=\sum_{j}\left(\beta_{j}^{m}\cdot {\bar{x}}_{j}/\mu \right)\cdot C_{j}+\sum_{j}\left(\gamma_{k}^{m}\cdot {\bar{z}}_{k}/\mu \right)\cdot C_{k}+GC_{u}/\mu$$ (4)

where *C*_*j*_ is the concentration index for a need factor *x*_*j*_, *C*_*k*_ is the concentration index for non- need factor *z*_*k*_, *μ* is the mean of the dependent variable and *GC*_*u*_ is the generalized concentration index for the error term. The marginal effect of each explanatory factor is evaluated at sample means, and $\beta_{j}^{m}\cdot {\bar{x}}_{j}/\mu$ and $\gamma_{k}^{m}\cdot {\bar{z}}_{k}/\mu$ represent the elasticity of each factor with the variable, and denoted as *η*_*j*_ or *η*_*k*_. Therefore, the contribution of each variable expresses the result of the product of the elasticity and the individual concentration index. A positive contribution can thus be the result of either two positive or two negative multipliers, while a negative contribution is the result of one negative and one positive multiplier.

Once we estimated the contributions for each need and non-need factor with this approach, the horizontal inequity index was then calculated by subtracting the contributions of the need factors from the unstandardised concentration index.

$$HII=C-C_{N}$$ (5)

An HII is interpreted similarly to the *C*, and its value also ranges from -1 to +1 [47], with a positive value indicating a distribution of healthcare access in favour of the rich, and vice versa, given similar healthcare needs [18]. *C*_*N*_ can be interpreted as the value for the concentration index if only need variables presented income-related inequalities or were the only variables associated to the utilisation variable.

In a final step, we decomposed the change in the *C* between periods, pre-crisis and crisis. Wagstaff demonstrated that the changes of *C* over time can be decomposed into the sum of changes in the contributions. Using an Oaxaca-type decomposition [45] we arrived at

$$\Delta C=\sum_{k}\eta_{k,t}\left(C_{k,t}-C_{k,t-1}\right)+\sum_{k}C_{k,t-1}\left(\eta_{k,t}-\eta_{k,t-1}\right)+\Delta GC_{u}$$ (6)

where sub-index *t-1* stands for the pre-crisis period and sub-index *t* for the crisis period. The first term in the equation indicates the distribution effect and measures the change in *C* caused by changes of the concentration indices of the explanatory variables *C*_*k*_ of *x*_*k*_. The second term of equation indicates to what extent a change of elasticity impacts on *C*.

Changes in the contribution of each variable were calculated this way and are reported individually. All analyses were initially performed separately for men and women and for both periods: pre-crisis and crisis. We found similar estimates in both sexes and, therefore, decided to collapse the data in order to achieve more robust estimations, and instead included sex as a need-variable. We used Stata software version 13.1.

## Results

Table 2 shows the characteristics of the study sample. Overall, utilisation of the four types of health services increased during the crisis compared to the pre-crisis period, with higher increments in hospital and emergency use (Table 2). With some exceptions, the distribution of the majority of the independent variables displayed slight changes between the pre-crisis (2007) and crisis (2011–12) periods. For example, the crisis period showed higher prevalence of poor mental health and accidents, but also a higher proportion of participants with secondary education. The most notable difference was the rise in unemployment, from 6.5% in the pre-crisis to 23.0% in the crisis period. The composite income index showed no variation between periods, nor was a major change detected in the percentage of participants with a private health insurance (around 7%). The distribution of participants by province and by size of municipality of residence was similar in both periods.

**Table 2:** Sample characteristics by period of study.

Variables		Period			
		2007		2011/12	
		n	%	n	%
Total		5011	100	5243	100
General practitioner consultation	Yes	1057	21.1	1194	22.8
	No	3954	78.9	4049	77.2
Specialist consultation	Yes	279	5.6	301	5.7
	No	4732	94.4	4942	94.3
Hospital admission	Yes	298	6.0	423	8.1
	No	4713	94.0	4820	91.9
Emergency attention	Yes	1025	20.5	1223	23.3
	No	3986	79.5	4020	76.7
Sex	0 = Men	2589	51.7	2656	50.7
	1 = Women	2422	48.3	2587	49.3
Age	25–44 years	2430	48.5	2439	46.5
	45–64 years	1664	33.2	1807	34.5
	65 and older	917	18.3	997	19.0
Self-rated health	0 = Good	3834	76.5	4044	77.1
	1 = Poor	1177	23.5	1199	22.9
Mental health	0 = Good	4014	80.1	3964	75.6
	1 = Poor	997	19.9	1279	24.4
Chronic conditions	None	2426	48.4	2709	51.7
	One	1191	23.8	1140	21.7
	Two or three	629	12.6	604	11.5
	Four or more	765	15.3	790	15.1
Accident	0 = No	4717	94.1	4857	92.6
	1 = Yes	294	5.9	386	7.4
Income	Lower than 1000€	604	12.1	1273	24.3
	1000 to 1499€	901	18.0	1603	30.6
	1500 to 1999€	675	13.5	696	13.3
	2000€ or higher	574	11.4	543	10.3
	Non response	2257	45.0	1128	21.5
Composite income index	Mean—Standard dev.	4.61	1.31	4.60	1.27
Education	No studies	693	13.8	719	13.7
	Up to 5 yr education	1147	22.9	1107	21.1
	Up to 8 yr education	1253	25.0	1346	25.7
	Secondary	1080	21.6	1334	25.4
	University	838	16.7	737	14.1
Private Insurance	0 = No	4643	92.7	4901	93.5
	1 = Yes	368	7.3	342	6.5
Size municipality	<10000	1059	21.1	1087	20.7
	10000 to 50000	1389	27.7	1509	28.8
	>50000	1064	21.2	1123	21.4
	province capital	1499	29.9	1524	29.1
Employment status	Working	2474	49.4	2023	38.6
	Unemployed	327	6.5	1204	23.0
	Retired	861	17.2	905	17.3
	Other	1349	26.9	1111	21.2
Province	Almería	393	7.8	439	8.4
	Cádiz	735	14.7	766	14.6
	Córdoba	499	10.0	504	9.6
	Granada	582	11.6	587	11.2
	Huelva	308	6.1	325	6.2
	Jaén	421	8.4	417	8.0
	Málaga	937	18.7	1025	19.5
	Sevilla	1136	22.7	1180	22.5

### Horizontal inequity before and during the crisis

**Fig 1:**
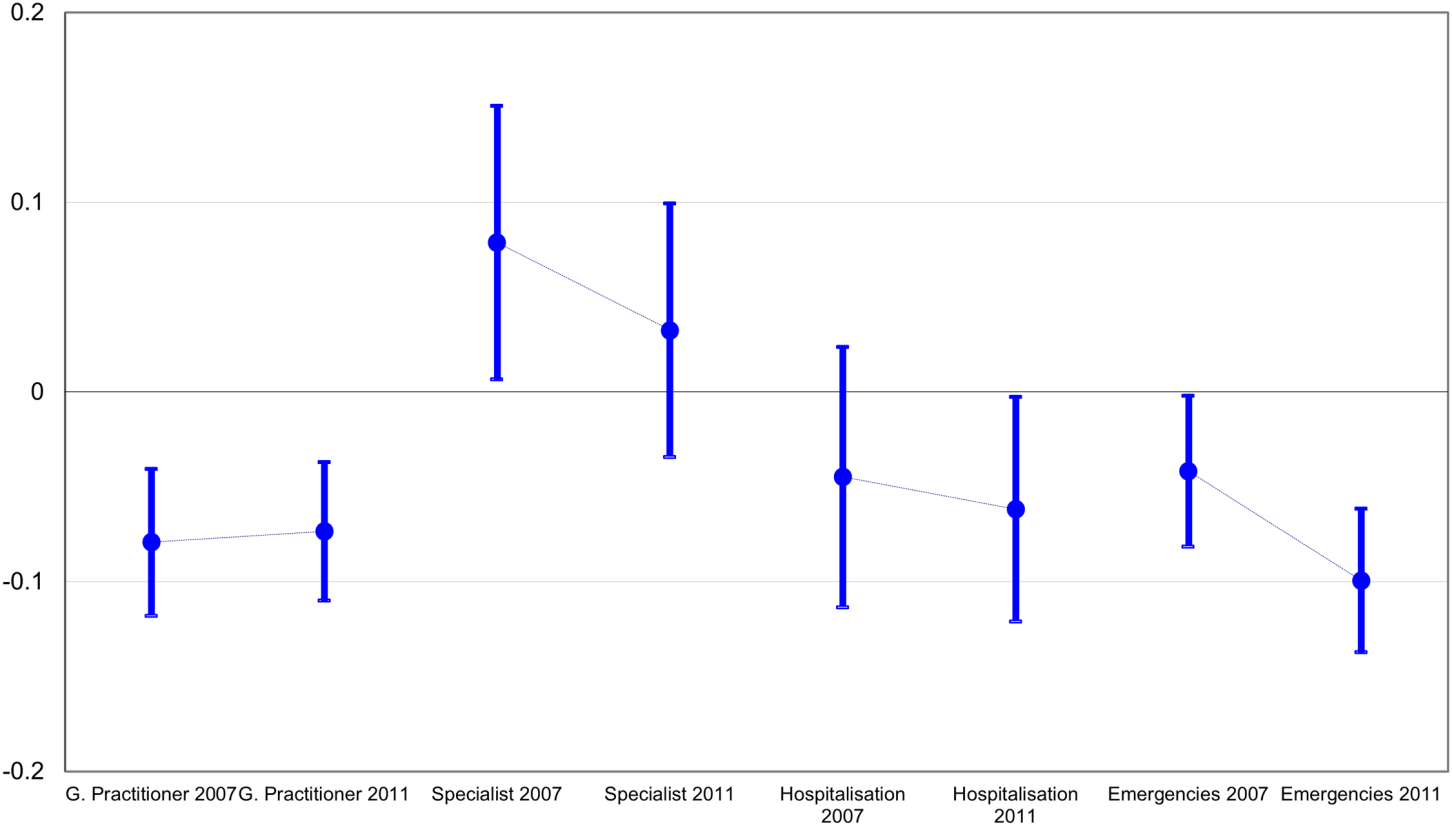
Horizontal inequity indices of income-related inequalities in health services utilisation by period. Andalusia, 2007 and 2011–2012.

Horizontal inequity differed depending on the type of service before the crisis (see Fig 1). Whereas GP consultations were concentrated among lower SES status persons (HII: -0.0792, CI95: -0.1178 –-0.0406), consultations with specialists were more common in the higher SES groups (HII: 0.0788, CI95: 0.0067–0.1509). Emergency ward use and hospitalisation (HII: -0.0448, CI95: -0.1134–0.0238) displayed smaller pro-poor inequities, significant only for emergency utilisation (HII: -0.0416, CI95: -0.0814 –-0.0018).

Inequity of health service use also changed between 2007 and 2011/2012 in different directions for different health services. Access to GP consultation changed marginally in the pro-rich direction, although remained decidedly in favour of low SES group. The other three types of health care services showed changes in the pro-poor direction. First, visits to specialists continued to be unequal in a pro-rich direction but no longer statistically significant. Second, hospitalisation changed to a significant pro-poor inequity. Third, emergency care presented the steepest change towards an even more pronounced inequity in favour of low SES group.

### Decomposing inequality in health care utilisation and the change in inequality between periods

**Fig 2:**
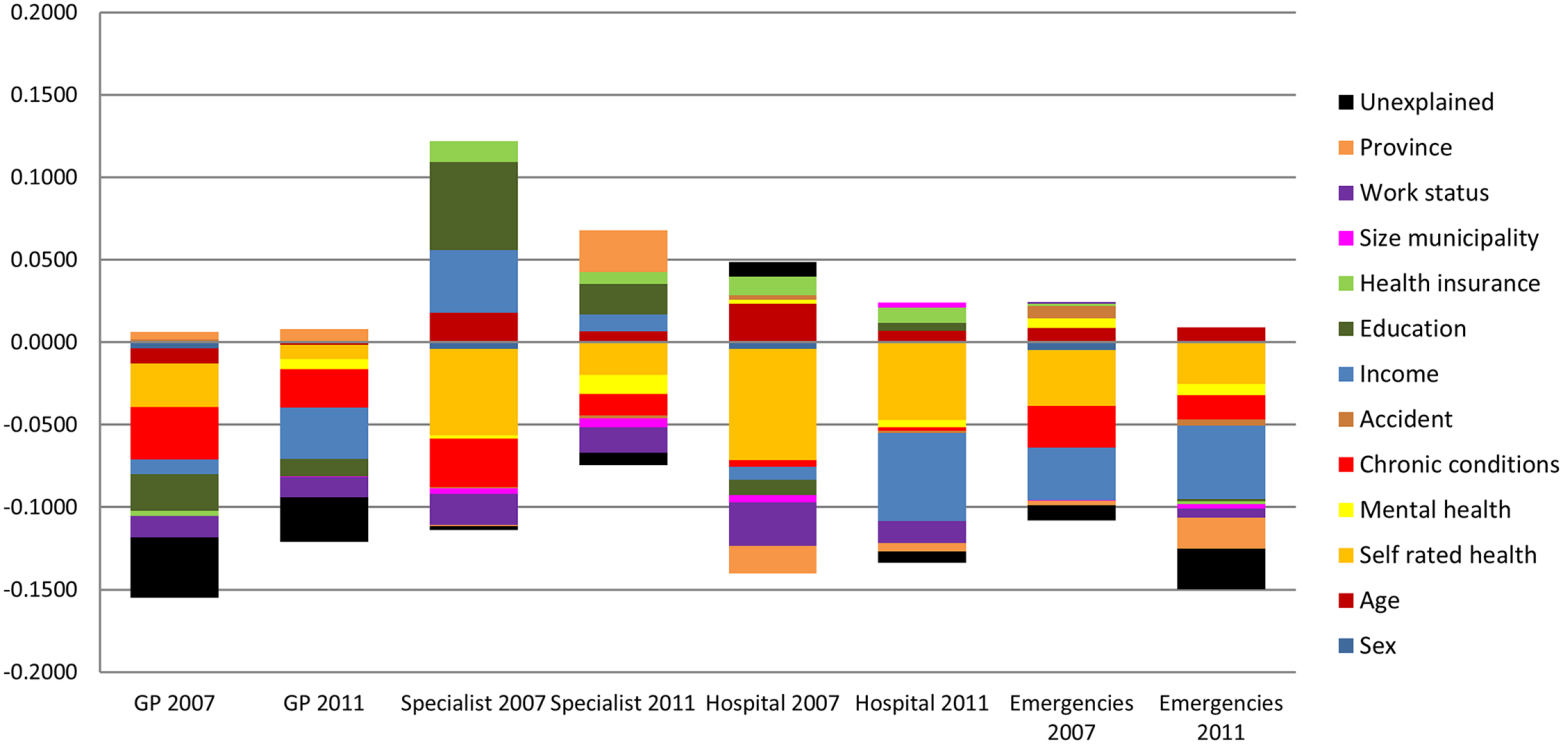
Decomposition of absolute contributions to the concentration indices of income-related inequalities in health services utilisation by period. Andalusia, 2007 and 2011–2012.

Prior to decomposing the change in the *C*, we analysed the distribution of the contributions of each variable to the overall *C* of the four healthcare use types, separately for the pre-crisis and crisis periods. This analysis thus illustrates possible contributing factors that remain stable between periods and whose contributions to *C* would not be detected in a simple analysis of the change. Fig 2 shows the contribution of each variable or factor to the *C*, that is, to the income related healthcare utilisation inequality, both in the pre-crisis and in the crisis period. Decomposition contributions, elasticities and concentation indices for each variable category by period are shown in S1 Table.

**Fig 3:**
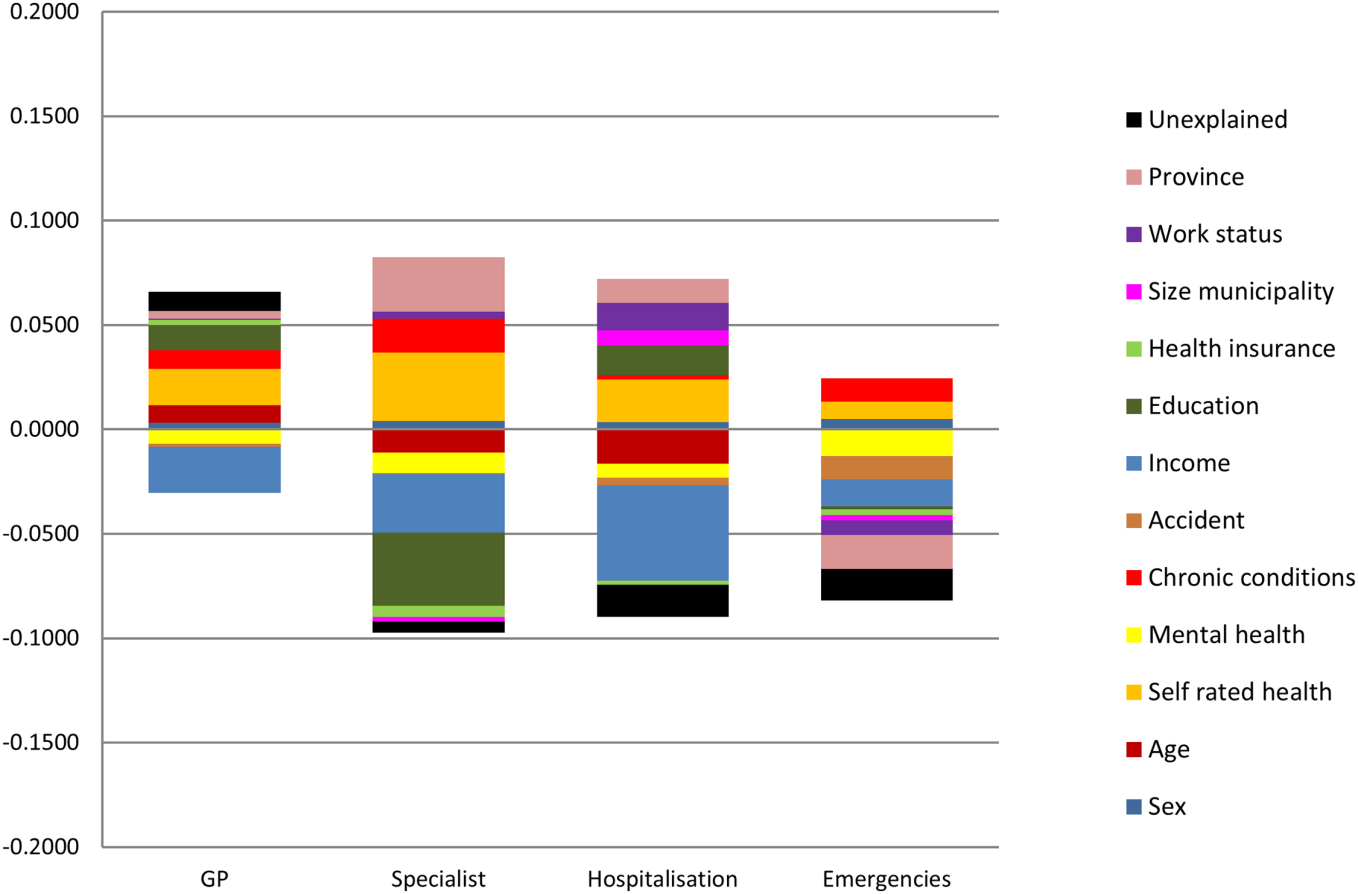
Period change (2007-2011/12) in absolute contributions to concentration indices of income related inequalities in health services utilization in Andalusia.

Corresponding to the second aim of this study, the change in income related inequalities in healthcare utilisation from pre-crisis to the crisis period was then decomposed. The period changes in the absolute contributions of each independent variable to concentration indices are shown in Fig 3. As notes in the Analysis subsection, the period change can be separated into two additive components, corresponding to the two terms of Eq (6): the first component is the change in the distribution (indicating the shift in the relationship between the variable and the *C*); and the second component is the change in the elasticity (which measures the change in the association between the independent variable and the outcome). Both components, as well as the total change in the contribution of each variable are shown in Table 3. The subsequent part of this paper moves on to describe in greater detail, separately for the four outcomes, (i) the pre-crisis contributions to *C* of each variable, and (ii) the contributions of each variable to the change in the *C* between periods. The change in the elasticity and in the distribution effect by variable category are shown in S2 Table.

**Table 3:** Period change (2007-2011/12) in elasticities, distribution effects and absolute contributions to concentration indices of income related inequalities in health services utilisation in Andalusia.

	General practitioner			Specialist			Hospitalisation			Emergency		
	Change in distribution	Change in elasticity	Change in contribution	Change in distribution	Change in elasticity	Change in contribution	Change in distribution	Change in elasticity	Change in contribution	Change in distribution	Change in elasticity	Change in contribution
**Sex**	0,0029	0,0003	0,0032	0,0009	0,0030	0,0039	0,0035	0,0000	0,0035	0,0023	0,0022	0,0045
**Age**	0,0003	0,0080	0,0083	-0,0057	-0,0054	-0,0112	-0,0100	-0,0063	-0,0163	-0,0111	0,0113	0,0002
**Self-rated health**	0,0014	0,0161	0,0176	0,0033	0,0296	0,0329	0,0079	0,0125	0,0205	0,0042	0,0043	0,0086
**Mental health**	0,0011	-0,0080	-0,0070	0,0019	-0,0118	-0,0098	0,0007	-0,0074	-0,0067	0,0012	-0,0138	-0,0127
**Chronic conditions**	0,0099	-0,0010	0,0088	0,0069	0,0092	0,0162	-0,0003	0,0021	0,0018	0,0050	0,0062	0,0112
**Accident**	0,0001	-0,0013	-0,0012	-0,0048	0,0042	-0,0006	-0,0035	-0,0003	-0,0038	-0,0123	0,0009	-0,0114
**Income**	0,0014	-0,0238	-0,0224	-0,0005	-0,0275	-0,0280	0,0024	-0,0481	-0,0457	0,0020	-0,0147	-0,0127
**Education**	0,0010	0,0110	0,0119	-0,0035	-0,0314	-0,0349	-0,0016	0,0158	0,0142	0,0000	-0,0015	-0,0015
**Health insurance**	0,0000	0,0026	0,0026	0,0006	-0,0059	-0,0054	0,0007	-0,0026	-0,0019	-0,0001	-0,0026	-0,0028
**Size of municipality**	-0,0014	0,0014	0,0001	-0,0043	0,0020	-0,0024	0,0005	0,0067	0,0073	-0,0048	0,0023	-0,0025
**Working status**	0,0019	0,0025	0,0006	0,0095	-0,0060	0,0034	0,0114	0,0064	0,0132	0,0012	-0,0081	-0,0069
**Province**	0,0077	-0,0071	0,0035	0,0159	0,0103	0,0261	-0,0021	0,0133	0,0115	-0,0053	-0,0113	-0,0162
**Unexplained**			0,0093			-0,0052			-0,0155			-0,0152
**CI**			0,0354			-0,0147			-0,0178			-0,0574

#### General practitioner consultations

Most variables contributed to GP use in a pro-poor direction in both periods, though some remarkable findings were detected regarding change in use of GP consultations. The main pro-poor change in the contribution to *C* was attributed to income, mostly due to a greater negative elasticity of this variable. This indicates that visits to GP became more concentrated in the lowest income population.

Concerning need variables, a change in the elasticity of poor mental health made a relevant pro-poor contribution in the change of the *C*, which indicates a stronger association of poor mental health and GP consultation in the second period. On the other hand, poor self-rated health was the most important contributor to a pro-rich change in GP use. This was due to the diminished association between poor self-rated health and GP consultations in the crisis period in combination with a persistent concentration of poor self-rated health among the lowest income groups. In contrast, the contribution to a pro-rich change of chronic diseases derived from the change in the distribution effect but not from the change in the elasticity.

Overall, although the change in *C* was clearly pro-rich, when we only consider the contributions of non-need variables, that is, HII, the estimated change was slightly positive, as we previously commented.

#### Specialist consultations

In the first period there was a predominant contribution of the non-need variables in a pro-rich direction, with income and higher educational level strongly associated with specialist visits. The observed change in contributions to inequality in the second period revealed that the socio-economic variables (income and education) clearly moved in a pro-poor direction, in relation to change in their elasticities.

Older age presented positive contributions in both periods as it showed a negative elasticity and was concentrated among the lowest income groups. Other need variables such as self-rated health and chronic conditions showed pro-poor contributions, but also displayed changes in a pro-rich direction due to the change in elasticity of the first one and to the concurrent change in elasticity and distribution effect in chronic conditions in the same direction. Poor mental health also contributed to a pro-poor change in utilisation based on the change of its elasticity.

#### Hospitalisations

In the pre-crisis period, the main contributor by far was poor self-rated health, which correlated with hospitalisations and concentrated among the lowest income population.

Concerning the change in inequality in hospital inpatient care between 2007 and 2011, the change in elasticity in income in a pro-poor direction was the major contributor, which was explained by a steep negative change in elasticity in relation to a great increase in utilisation of the two lowest quintiles of income. The negative change in the contribution of age was also remarkable, associated to a change in the distribution effect in the younger, indicating that higher income was less concentrated in the 25–44 years group in the second period while elasticity remained steadily positive. These negative contributions were however off-set by positive changes in self-rated health and education, both of them due to change in elasticities.

#### Emergency attentions

Income and need variables showed pro-poor contributions in the first period. Regarding the change in inequality in emergency attentions, where the overall greatest pro-poor distribution in the crisis period was observed, no predominant role of any need or non-need variable was found. Both mental health, in relation to change in elasticities (poor mental health was more related to emergency attentions during the crisis), and accidents, in relation to change in the distribution of the concentration index (accidents shifted to concentrate among the lower income adults during the crisis), showed relevant contributions in the pro-poor direction. Also income and province of residence presented negative changes in their contributions. On the contrary, pro-rich contributions, though moderate, relied on self-rated health and chronic conditions, similarly to what happened in the other outcomes variables.

We provide the joint contributions to change by variable groups in Table 4. In summary, need variables (self-rated health, chronic conditions, mental health and accidents) contributions to the change in *C* were pro-rich in three of the four outcomes whereas the overall contributions of non-need socioeconomic variables (income, education, insurance and working status) were consistently in the pro-poor direction. Contributions of demographic (age and sex) and non-need geographical variables (province and size of municipality) to change in *C* were rather inconsistent. Finally, unexplained contributions were negative in most service utilisations, higher for hospital admissions and emergency attentions.

**Table 4:** Summarized period change (2007-2011/12) contributions to concentration indices of income related inequalities in health services utilisation in Andalusia.

Variable group	General practitioner	Specialist	Hospital admission	Emergency attention
**Demographic**	0.0115	-0.0073	-0.0127	0.0047
**Need**	0.0182	0.0387	0.0118	-0.0044
**Non-need socioeconomic**	-0.0072	-0.0648	-0.0202	-0.0239
**Non-need geographical**	0.0035	0.0238	0.0188	-0.0187
**Unexplained**	0.0093	-0.0052	-0.0155	-0.0152
**Concentration Index (*C*)**	0.0354	-0.0147	-0.0178	-0.0574

## Discussion

The present study set out to illustrate the impact of the current financial crisis on social inequities in the utilisation of multiple forms of healthcare, as well as to assess these changes in inequity, in a context hit hard by the crisis. Our results show that during the first years of the economic recession in Andalusia, the use of several important health services did not decrease, and needs-adjusted socioeconomic inequities in utilisation of these services either narrowed (GP and specialist consultations) or increased in a pro-poor direction (hospital and emergency care). Decomposition analysis indicated that socioeconomic conditions and, to a lesser degree, poor mental health explained a considerable portion of the pro-poor change in inequality in healthcare utilisation. Meanwhile self-rated health and chronic conditions were the main contributors in a pro-rich direction. Most changes in contributions were attributable to modification in the association of the variables with the utilisation outcomes, but not with income-related redistribution of the factors.

Hitherto, findings concerning the impact of the economic crisis on inequities in the use of health services throughout Europe are scarce and inconsistent [48,49]. Even less is known on the underlying factors explaining these inequities. To our knowledge this is one of the few publications in the public health domain on decomposing the change in inequality [45,50,51] and the first to do so evaluating the potential impact of the economic recession.

### General practitioner consultations

The meagre change observed in the inequality of use of GP consultations is not a surprising observation. In a study performed in Spain in 2006–2007 in population aged 50 and older, Crespo et al. detected pro-poor inequality in access to GPs, which was attributable mainly to an unequal distribution of need factors [52]. In our case, the role of need-factors contribution is remarkable in both periods. They also contributed to the change in inequalities in a pro-rich direction, whereas the contributions of income and poor mental health were in a pro-poor direction.

### Specialist consultations

Regarding specialist visits, we detected an unexpected reduction in pro-rich inequity. This finding is in contrast with the previously cited study by García Subirats et al. [48], who reported increased pro-rich social class inequalities in specialist visits from 2006 to 2011 in the non-migrant Spanish population. Using the same databases during the same period, another study showed that people in lower classes tended to increase their access to GPs and reduced their access to specialist care [53]. However, our findings are consistent with those of Abásolo et al. [23], who compared separately public and private healthcare use by income quartiles before and after the onset of the crisis in Spain, and showed that financial crisis was related to a decrease in utilisation of private specialists among highest income groups.

In our study, the main contributors to the pro-poor changes in healthcare utilization were income and education. This was explained by a remarkable reduction in their elasticities, as higher income and especially better educated groups used proportionally less specialist services in the crisis period. Age also contributed in a pro-poor direction with a change in in the distribution effect in the younger. This finding is consistent with previous research highlighting that higher income was less concentrated among young adults in Spain [13]. Poor mental health was the main need contributor to the pro-poor in the contribution to *C* for specialist use.

It is important to point out that our study included the use of both private and public outpatient specialist medical services. We therefore cannot discriminate if the utilisation of out-of-pocket private services might have been reduced in these higher income and education groups in the first stages of the crisis. From the perspective of public health policies, it is more relevant to reduce inequalities in the use of public services, considering that people with higher incomes have greater access to private services (out-of-pocket or health insurance) in the context of a mixed public/private provision health system.

### Hospitalisations

Our finding of moderately increasing horizontal inequity in hospital admissions in favour of the less-advantaged groups contrasts to a previous study at the national level, which reported no significant inequality in the access to hospitalisation and in associated factors in relation with the economic recession [48]. Among the scarce literature on change in hospitalisations during recessions, a study performed in Finland during the crisis of the middle 1990s described a slight pro-rich increase in inequality, although health system’s characteristics, like co-payments for inpatient services, could limit the comparability of both studies [54]. More recently, Urbanos and González reported a non-significant reduction in the odds of hospitalisation of lower classes compared to upper classes (manual versus non-manual) in Spain between 2006 and 2011 [22].

An increase in hospitalisations in the more deprived groups is not a positive development in itself, since, in a context of universal health coverage, it could indicate a deterioration in the health status in these groups. In this vein, the change in inequality in hospital admissions in our study was related to a rise in hospital admissions in the two lowest quintiles of income in the sample. A review on the short term impact of the current economic crisis on health and health care use indeed showed that in countries with moderate cuts to health expenditure, higher unemployment was associated with increased hospital admission rates [55], though the study shed no light regarding socio-economic inequalities in hospital use. Additionally, we found no data to attribute this increase to the compensation for the lack of access of these groups to any other type of healthcare.

### Emergency attentions

Concerning inequalities in emergency attentions, we demonstrated changes to a significantly pro-poor inequity in the second period. In a specific study on emergencies during the crisis period in Spain that did not specifically examine inequalities, poor mental health and limitations for activities in daily living were related to an increase in access, after controlling for socio-demographic and health variables [56]. The already mentioned study in Spain detected an increase in emergency care utilisation by lower social classes compared to higher classes the previous year [22]. Another study from Spain also showed a greater pro-poor distribution of emergency attentions use after the onset of the economic crisis [23]. These results are comparable to what we observed in our study in Andalusia; an increase in pro-poor inequality in the use of emergencies, which was related to a stronger relationship of poor mental health and emergency attentions and to a greater concentration of accidents among the lower income groups during the crisis.

### Overall interpretation

At large, for the four outcomes we consistently observed that contributions of self-rated health were pro-rich due to changes in elasticities, indicating a change to a weaker association between poor self-rated health and service use in the crisis period. It is possible that self-rated health is not performing as good a predictor of use of healthcare service in times of the economic crisis. A recent study on time trends of self-rated health in European countries indeed reported a weaker association between this measure and chronic diseases over time, possibly due to people attaching significance to different aspects of health when rating their self-rated health [57]. This could also explain, for instance, why several studies have shown an increase in the prevalence of good self-rated health during the current economic recession compared to previous periods in different settings, including Spain [58]. We hypothesise that people with a similar level of health perceived a better health in the second period, thus reducing the elasticity of self-rated health in relation to the healthcare use outcomes.

Poor mental health was associated to pro-poor inequality in the use of services, in relation to increased elasticity in the four outcomes. This was attributable both to a higher prevalence of poor mental health in the crisis period, and to a stronger association of poor mental with service use, together with a consistent concentration of this condition among the lower income groups. As such, we may hypothesise that men and women in Andalusia have been more exposed to mental health problems related to unemployment and to the fear of job loss in the second period [59], and might have, in consequence, increased their demands of use of all types of healthcare services included in this study. In summary, pro-poor changes in all outcomes were related to higher concentration of use among people in lower income groups, and a stronger relationship of poor mental health with healthcare use during the crisis. Pro-poor changes were partially offset by pro-rich contributions of self-rated health, which was less associated to healthcare use in the second period, and also by a comparatively greater concentration on chronic diseases in higher income groups in this period. However, changes in distribution effects, that is to say, in income-related redistribution of need and non-need factors, barely explained the changes in the contributions of young age in several utilisation outcomes.

The reductions of inequities and the pro-poor direction of changes found for most healthcare types could reflect a buffering role of a public health system with universal health coverage against the detrimental effects of the crisis. This withstanding capacity seemingly has been retained despite the imposed restrictions in budgets, mainly executed by reducing staff and transferring some costs to the people. Despite considerable cuts in the health system’s finances, the principle of solidarity was not seriously shaken, at the same time that structural reforms were relatively mild compared to other countries, such as the United Kingdom [60].

Though these statements can be applied to the first years of the economic recession, some hints of the plausible speed-up in the deterioration of health services after a period of compensation and adequate response of the health system have been detected. After what could be named “doing the same with less”, there is a certain risk of a major increase in access barriers and decrease in the quality of services, leading to genuine harm to patients due to prolonged austerity policies [61].

This further is supported in qualitative studies based on the opinions of physicians, showing their concern for how much the system can resist in the long run [62]. Moreover, empirical data from Spain reveals a rapid increase in the number of patients in surgical waiting lists since the middle of 2011 and a threefold rise in the rates of the population unsatisfied with the performance of the health system between 2011 and 2014 [63].

### Limitations of the study

We note that the cross-sectional design does not enable us to analyse associations in terms of causality. Moreover, it is plausible that the data source did not identify inequalities in health services use of groups more frequently underrepresented in population health studies based on household surveys, such as migrants, young adults and people facing eviction or at risk of social exclusion. Some of these conditions were more prevalent in the crisis period and also related to socio-economic inequalities, and thus this situation could have introduced some bias in our results in case they were also related to differential service access rates. On the other hand, the share of the total healthcare utilisation corresponding to these subgroups is low and thus with limited impact in our results.

We did not distinguish between utilisation of private and public services. The proportion of attentions received in private settings might be considerable for specialist visits and hospitalisation. Our data source did not include this distinction. The inclusion of the variable private insurance in the analysis may partially account for this missing information, as private insurance holders are known to use private services more frequently than people without private insurance coverage.

Our outcome variables are widely used in the health services and inequalities literature but they only evaluate the probability of contact with a health service, thus ignoring a plausible deterioration in the quality of performance due to reduction in healthcare personnel or to an increase in waiting times for surgery, GP or specialist consultation as reported in different settings [64–66]. Indicators of quality of service usually are not available on population health surveys, and this poses a limitation that requires further investigation in other studies.

Finally, though the composite index that we have used as a proxy to income to rank our sample by socio-economic status has not been externally validated, we are confident with its performance as a rank variable according to the internal validity and the high correlation with available socio-economic data.

## Conclusion

This study expands the knowledge of the changes in inequity in healthcare utilisation associated with the economic crisis and ensuing austerity measures, in a context of a universal coverage health system in Southern Europe. Our results show that during the first years of the economic recession in Andalusia, the use of relevant health services did not decrease, and needs-adjusted socioeconomic inequities in utilisation of these services were reduced, GP and specialist consultations, or moved in a pro-poor direction as was the case for hospital and emergency attentions.

The study also provides additional evidence with respect to the underlying factors associated to these modifications. Decomposition analysis indicated that socioeconomic conditions and, to a lesser degree, poor mental health explained a remarkable proportion of the pro-poor change in inequality in service utilisation, while self-rated health and chronic conditions were the main contributors in a pro-rich direction both before and during the crisis. The majority of changes in the contributions to inequality were attributable to changed associations with utilisation, and not with socioeconomic-related redistribution of the variables between the two periods. This finding also supports the idea that the universal health system has steadily performed favouring equity of healthcare utilisation despite budget cuts and resource constraints. However, a greater use of certain services such as emergencies or hospitalisations by lower socioeconomic groups could be indicating a limited access to appropriate primary care services. Thus, further research is needed to monitor the potential impact of restrictive policies in the coming years. We conclude that despite the withstanding capacity displayed during the first years of the recession, efforts have to be made to ensure equity in the performance of the health system in Andalusia, especially if new research continues to associate the economic recession with deleterious health effects.

## Supporting information

S1 File Stata commands (.do file) for the study. (DO)

S1 Table Decomposition contributions, elasticities and concentration indices by period. (DOC)

S2 Table Change in elasticity and distribution effect by variable category. (DOC)
