# Supplementary material for: Withstanding austerity: Equity in health services utilisation in the first stage of the economic recession in Southern Spain
Source: PLoS One. 2018 Mar 30;13(3):e0195293. doi: 10.1371/journal.pone.0195293 (PMC5877882; doi:10.1371/journal.pone.0195293)
Supplement: S1 Table — (DOC) [file pone.0195293.s002.doc]

**S1 Table**

Decomposition contributions, elasticities and concentration indices by period.

|  | 1A. General practitioner | | | | | | | | |
| --- | --- | --- | --- | --- | --- | --- | --- | --- | --- |
|  | Pre-crisis | | | |  | Crisis | | | |
|  | elast | ci | contrib | percent |  | elast | ci | contrib | percent |
| Sex | 0,4424 | -0,0082 | -0,0036 | 0,0244 |  | 0,4013 | -0,0011 | -0,0004 | 0,0039 |
| Young | 0,0451 | 0,0611 | 0,0028 | -0,0186 |  | 0,0107 | 0,0199 | 0,0002 | -0,0019 |
| Old | 0,0436 | -0,2804 | -0,0122 | 0,0824 |  | 0,0076 | -0,1797 | -0,0014 | 0,0120 |
| Poor self rated health | 0,1292 | -0,2019 | -0,0261 | 0,1757 |  | 0,0494 | -0,1726 | -0,0085 | 0,0754 |
| Poor mental health | -0,0047 | -0,1276 | 0,0006 | -0,0040 |  | 0,0583 | -0,1094 | -0,0064 | 0,0564 |
| One chronic condition | 0,1237 | 0,0320 | 0,0040 | -0,0267 |  | 0,1143 | 0,0357 | 0,0041 | -0,0361 |
| Two-three chronic conditions | 0,1087 | -0,1053 | -0,0114 | 0,0771 |  | 0,0836 | -0,0419 | -0,0035 | 0,0310 |
| Four or more chronic conditions | 0,1259 | -0,1938 | -0,0244 | 0,1644 |  | 0,1434 | -0,1648 | -0,0236 | 0,2091 |
| Accident | 0,0211 | 0,0577 | 0,0012 | -0,0082 |  | -0,0007 | -0,0257 | 0,0000 | -0,0002 |
| Income | -0,0556 | 0,1607 | -0,0089 | 0,0602 |  | -0,2036 | 0,1537 | -0,0313 | 0,2770 |
| Up to 5 yr education | 0,0293 | -0,1323 | -0,0039 | 0,0261 |  | -0,0172 | -0,1108 | 0,0019 | -0,0168 |
| Up to 8 yr education | -0,0080 | 0,0335 | -0,0003 | 0,0018 |  | -0,0185 | -0,0067 | 0,0001 | -0,0011 |
| Secondary studies | -0,0381 | 0,1056 | -0,0040 | 0,0271 |  | -0,0258 | 0,0899 | -0,0023 | 0,0205 |
| University studies | -0,0505 | 0,2813 | -0,0142 | 0,0957 |  | -0,0368 | 0,2756 | -0,0101 | 0,0897 |
| Health insurance | -0,0103 | 0,2737 | -0,0028 | 0,0189 |  | -0,0007 | 0,2961 | -0,0002 | 0,0018 |
| Municipality pop. 10 to 50,000 | 0,0173 | 0,0288 | 0,0005 | -0,0034 |  | 0,0311 | 0,0120 | 0,0004 | -0,0033 |
| Municipality pop. > 50000 | 0,0051 | -0,0319 | -0,0002 | 0,0011 |  | 0,0016 | -0,1154 | -0,0002 | 0,0016 |
| Province capital | 0,0140 | -0,0407 | -0,0006 | 0,0038 |  | -0,0081 | 0,0459 | -0,0004 | 0,0033 |
| Unemployed | 0,0076 | -0,1689 | -0,0013 | 0,0086 |  | 0,0244 | -0,1701 | -0,0041 | 0,0367 |
| Retired | 0,0604 | -0,1941 | -0,0117 | 0,0790 |  | 0,0323 | -0,1359 | -0,0044 | 0,0388 |
| Other | -0,0016 | -0,0467 | 0,0001 | -0,0005 |  | 0,0012 | -0,0031 | -0,0038 | 0,0000 |
| Cádiz | -0,0077 | -0,1882 | 0,0015 | -0,0098 |  | 0,0128 | -0,2872 | -0,0037 | 0,0325 |
| Córdoba | 0,0272 | 0,0589 | 0,0016 | -0,0108 |  | 0,0401 | 0,1737 | 0,0070 | -0,0616 |
| Granada | 0,0262 | 0,0761 | 0,0020 | -0,0134 |  | -0,0146 | -0,0199 | 0,0003 | -0,0026 |
| Huelva | 0,0184 | 0,0000 | 0,0004 | -0,0030 |  | 0,0068 | 0,0233 | 0,0002 | -0,0014 |
| Jaén | 0,0144 | 0,0137 | 0,0002 | -0,0013 |  | 0,0250 | 0,2006 | 0,0050 | -0,0444 |
| Málaga | 0,1030 | -0,0702 | -0,0072 | 0,0487 |  | 0,0276 | -0,0257 | -0,0007 | 0,0063 |
| Sevilla | 0,0716 | 0,0846 | 0,0061 | -0,0408 |  | -0,0003 | 0,0942 | 0,0000 | 0,0003 |
| Explained |  |  | -0,1120 |  |  |  |  | -0,0859 |  |
| Unexplained |  |  | -0,0364 |  |  |  |  | -0,0271 |  |
| CI |  |  | -0,1485 |  |  |  |  | -0,1130 |  |

|  | 1B. Specialist | | | | | | | | |
| --- | --- | --- | --- | --- | --- | --- | --- | --- | --- |
|  | Pre-crisis | | | |  | Crisis | | | |
|  | elast | ci | contrib | percent |  | elast | ci | contrib | percent |
| Sex | 0,4922 | -0,0082 | -0,0040 | -0,4981 |  | 0,1287 | -0,0011 | -0,0001 | 0,0213 |
| Young | 0,0317 | 0,0611 | 0,0019 | 0,2389 |  | 0,0648 | 0,0199 | 0,0013 | -0,1947 |
| Old | -0,0568 | -0,2804 | 0,0159 | 1,9671 |  | -0,0302 | -0,1797 | 0,0054 | -0,8201 |
| Poor self rated health | 0,2595 | -0,2019 | -0,0524 | -6,4673 |  | 0,1128 | -0,1726 | -0,0195 | 2,9394 |
| Poor mental health | 0,0146 | -0,1276 | -0,0019 | -0,2300 |  | 0,1068 | -0,1094 | -0,0117 | 1,7633 |
| One chronic condition | 0,0585 | 0,0320 | 0,0019 | 0,2309 |  | 0,0796 | 0,0357 | 0,0028 | -0,4286 |
| Two-three chronic conditions | 0,1088 | -0,1053 | -0,0115 | -1,4133 |  | 0,0682 | -0,0419 | -0,0029 | 0,4318 |
| Four or more chronic conditions | 0,1023 | -0,1938 | -0,0198 | -2,4471 |  | 0,0802 | -0,1648 | -0,0132 | 1,9962 |
| Accident | -0,0157 | 0,0577 | -0,0009 | -0,1121 |  | 0,0571 | -0,0257 | -0,0015 | 0,2215 |
| Income | 0,2372 | 0,1607 | 0,0381 | 4,7045 |  | 0,0661 | 0,1537 | 0,0102 | -1,5344 |
| Up to 5 yr education | 0,1259 | -0,1323 | -0,0166 | -2,0546 |  | 0,0936 | -0,1108 | -0,0104 | 1,5660 |
| Up to 8 yr education | 0,1664 | 0,0335 | 0,0056 | 0,6888 |  | 0,0990 | -0,0067 | -0,0007 | 0,0996 |
| Secondary studies | 0,1570 | 0,1056 | 0,0166 | 2,0460 |  | 0,0656 | 0,0899 | 0,0059 | -0,8906 |
| University studies | 0,1693 | 0,2813 | 0,0476 | 5,8795 |  | 0,0850 | 0,2756 | 0,0234 | -3,5344 |
| Health insurance | 0,0468 | 0,2737 | 0,0128 | 1,5797 |  | 0,0250 | 0,2961 | 0,0074 | -1,1191 |
| Municipality pop. 10 to 50,000 | -0,0512 | 0,0288 | -0,0015 | -0,1816 |  | 0,0053 | 0,0120 | 0,0001 | -0,0095 |
| Municipality pop. > 50000 | 0,0289 | -0,0319 | -0,0009 | -0,1137 |  | 0,0498 | -0,1154 | -0,0057 | 0,8678 |
| Province capital | 0,0240 | -0,0407 | -0,0010 | -0,1205 |  | -0,0009 | 0,0459 | 0,0000 | 0,0064 |
| Unemployed | -0,0155 | -0,1689 | 0,0026 | 0,3234 |  | -0,0133 | -0,1701 | 0,0023 | -0,3403 |
| Retired | 0,1015 | -0,1941 | -0,0197 | -2,4320 |  | 0,1274 | -0,1359 | -0,0173 | 2,6130 |
| Other | 0,0330 | -0,0467 | -0,0015 | -0,1904 |  | 0,0468 | -0,0031 | -0,0001 | 0,0217 |
| Cádiz | -0,0394 | -0,1882 | 0,0074 | 0,9149 |  | -0,0550 | -0,2872 | 0,0158 | -2,3867 |
| Córdoba | -0,0565 | 0,0589 | -0,0033 | -0,4103 |  | 0,0285 | 0,1737 | 0,0049 | -0,7464 |
| Granada | -0,0535 | 0,0761 | -0,0041 | -0,5026 |  | 0,0078 | -0,0199 | -0,0002 | 0,0233 |
| Huelva | -0,0242 | 0,0000 | -0,0006 | -0,0001 |  | 0,0058 | 0,0233 | 0,0001 | -0,0205 |
| Jaén | -0,0380 | 0,0137 | -0,0005 | -0,0642 |  | 0,0301 | 0,2006 | 0,0060 | -0,9123 |
| Málaga | -0,0792 | -0,0702 | 0,0056 | 0,6858 |  | 0,0504 | -0,0257 | -0,0013 | 0,1956 |
| Sevilla | -0,0644 | 0,0846 | -0,0055 | -0,6728 |  | -0,0036 | 0,0942 | -0,0003 | 0,0511 |
| Explained |  |  | 0,0103 |  |  |  |  | 0,0008 |  |
| Unexplained |  |  | -0,0022 |  |  |  |  | -0,0074 |  |
| CI |  |  | 0,0081 |  |  |  |  | -0,0066 |  |

|  | 1C. Hospitalisation | | | | | | | | |
| --- | --- | --- | --- | --- | --- | --- | --- | --- | --- |
|  | Pre-crisis | | | |  | Crisis | | | |
|  | elast | ci | contrib | percent |  | elast | ci | contrib | percent |
| Sex | 0,4991 | -0,0082 | -0,0041 | 0,0446 |  | 0,4962 | -0,0011 | -0,0005 | 0,0050 |
| Young | 0,2238 | 0,0611 | 0,0137 | -0,1491 |  | 0,2012 | 0,0199 | 0,0040 | -0,0365 |
| Old | -0,0344 | -0,2804 | 0,0096 | -0,1050 |  | -0,0168 | -0,1797 | 0,0030 | -0,0276 |
| Poor self rated health | 0,3329 | -0,2019 | -0,0672 | 0,7325 |  | 0,2708 | -0,1726 | -0,0467 | 0,4264 |
| Poor mental health | -0,0193 | -0,1276 | 0,0025 | -0,0268 |  | 0,0388 | -0,1094 | -0,0042 | 0,0387 |
| One chronic condition | -0,0039 | 0,0320 | -0,0001 | 0,0014 |  | 0,0254 | 0,0357 | 0,0009 | -0,0082 |
| Two-three chronic conditions | 0,0024 | -0,1053 | -0,0003 | 0,0028 |  | -0,0169 | -0,0419 | 0,0007 | -0,0065 |
| Four or more chronic conditions | 0,0194 | -0,1938 | -0,0037 | 0,0409 |  | 0,0236 | -0,1648 | -0,0039 | 0,0355 |
| Accident | 0,0470 | 0,0577 | 0,0027 | -0,0296 |  | 0,0417 | -0,0257 | -0,0011 | 0,0098 |
| Income | -0,0489 | 0,1607 | -0,0079 | 0,0856 |  | -0,3481 | 0,1537 | -0,0535 | 0,4882 |
| Up to 5 yr education | -0,0418 | -0,1323 | 0,0055 | -0,0603 |  | 0,0470 | -0,1108 | -0,0052 | 0,0475 |
| Up to 8 yr education | -0,0484 | 0,0335 | -0,0016 | 0,0177 |  | 0,0386 | -0,0067 | -0,0003 | 0,0023 |
| Secondary studies | -0,0610 | 0,1056 | -0,0064 | 0,0702 |  | 0,0639 | 0,0899 | 0,0057 | -0,0524 |
| University studies | -0,0248 | 0,2813 | -0,0070 | 0,0759 |  | 0,0159 | 0,2756 | 0,0044 | -0,0400 |
| Health insurance | 0,0413 | 0,2737 | 0,0113 | -0,1230 |  | 0,0317 | 0,2961 | 0,0094 | -0,0857 |
| Municipality pop. 10 to 50,000 | 0,0498 | 0,0288 | 0,0014 | -0,0156 |  | -0,0356 | 0,0120 | -0,0004 | 0,0039 |
| Municipality pop. > 50000 | 0,0690 | -0,0319 | -0,0022 | 0,0240 |  | -0,0478 | -0,1154 | 0,0055 | -0,0503 |
| Province capital | 0,0873 | -0,0407 | -0,0036 | 0,0387 |  | -0,0467 | 0,0459 | -0,0021 | 0,0196 |
| Unemployed | 0,0309 | -0,1689 | -0,0052 | 0,0569 |  | -0,0036 | -0,1701 | 0,0006 | -0,0057 |
| Retired | 0,0961 | -0,1941 | -0,0187 | 0,2032 |  | 0,1012 | -0,1359 | -0,0138 | 0,1255 |
| Other | 0,0544 | -0,0467 | -0,0025 | 0,0277 |  | 0,0208 | -0,0031 | -0,0001 | 0,0006 |
| Cádiz | 0,0661 | -0,1882 | -0,0124 | 0,1355 |  | -0,0018 | -0,2872 | 0,0005 | -0,0046 |
| Córdoba | -0,0160 | 0,0589 | -0,0009 | 0,0102 |  | 0,0125 | 0,1737 | 0,0022 | -0,0199 |
| Granada | -0,0080 | 0,0761 | -0,0006 | 0,0066 |  | -0,0218 | -0,0199 | 0,0004 | -0,0039 |
| Huelva | 0,0324 | 0,0000 | 0,0008 | -0,0085 |  | -0,0141 | 0,0233 | -0,0003 | 0,0030 |
| Jaén | 0,0482 | 0,0137 | 0,0007 | -0,0072 |  | -0,0167 | 0,2006 | -0,0034 | 0,0306 |
| Málaga | 0,0960 | -0,0702 | -0,0067 | 0,0734 |  | -0,0396 | -0,0257 | 0,0010 | -0,0093 |
| Sevilla | 0,0291 | 0,0846 | 0,0025 | -0,0269 |  | -0,0611 | 0,0942 | -0,0058 | 0,0525 |
| Explained |  |  | -0,1006 |  |  |  |  | -0,1029 |  |
| Unexplained |  |  | 0,0088 |  |  |  |  | -0,0067 |  |
| CI |  |  | -0,0918 |  |  |  |  | -0,1096 |  |

|  | 1D. Emergency | | | | | | | | |
| --- | --- | --- | --- | --- | --- | --- | --- | --- | --- |
|  | Pre-crisis | | | |  | Crisis | | | |
|  | elast | ci | contrib | percent |  | elast | ci | contrib | percent |
| Sex | 0,5951 | -0,0082 | -0,0049 | 0,0583 |  | 0,3238 | -0,0011 | -0,0004 | 0,0025 |
| Young | 0,1567 | 0,0611 | 0,0096 | -0,1146 |  | 0,2048 | 0,0199 | 0,0041 | -0,0289 |
| Old | 0,0036 | -0,2804 | -0,0010 | 0,0120 |  | -0,0263 | -0,1797 | 0,0047 | -0,0335 |
| Poor self rated health | 0,1661 | -0,2019 | -0,0335 | 0,4012 |  | 0,1446 | -0,1726 | -0,0250 | 0,1770 |
| Poor mental health | -0,0449 | -0,1276 | 0,0057 | -0,0685 |  | 0,0634 | -0,1094 | -0,0069 | 0,0492 |
| One chronic condition | 0,0942 | 0,0320 | 0,0030 | -0,0360 |  | 0,0530 | 0,0357 | 0,0019 | -0,0134 |
| Two-three chronic conditions | 0,0668 | -0,1053 | -0,0070 | 0,0840 |  | 0,0342 | -0,0419 | -0,0014 | 0,0102 |
| Four or more chronic conditions | 0,1113 | -0,1938 | -0,0216 | 0,2578 |  | 0,0903 | -0,1648 | -0,0149 | 0,1055 |
| Accident | 0,1322 | 0,0577 | 0,0076 | -0,0913 |  | 0,1480 | -0,0257 | -0,0038 | 0,0269 |
| Income | -0,1983 | 0,1607 | -0,0319 | 0,3809 |  | -0,2900 | 0,1537 | -0,0446 | 0,3161 |
| Up to 5 yr education | 0,0099 | -0,1323 | -0,0013 | 0,0156 |  | 0,0259 | -0,1108 | -0,0029 | 0,0204 |
| Up to 8 yr education | 0,0027 | 0,0335 | 0,0001 | -0,0011 |  | 0,0172 | -0,0067 | -0,0001 | 0,0008 |
| Secondary studies | 0,0221 | 0,1056 | 0,0023 | -0,0279 |  | -0,0115 | 0,0899 | -0,0010 | 0,0074 |
| University studies | -0,0037 | 0,2813 | -0,0010 | 0,0124 |  | 0,0095 | 0,2756 | 0,0026 | -0,0186 |
| Health insurance | 0,0043 | 0,2737 | 0,0012 | -0,0140 |  | -0,0054 | 0,2961 | -0,0016 | 0,0112 |
| Municipality pop. 10 to 50,000 | 0,0975 | 0,0288 | 0,0028 | -0,0335 |  | 0,0419 | 0,0120 | 0,0005 | -0,0036 |
| Municipality pop. > 50000 | 0,0656 | -0,0319 | -0,0021 | 0,0250 |  | 0,0144 | -0,1154 | -0,0017 | 0,0118 |
| Province capital | 0,0219 | -0,0407 | -0,0009 | 0,0106 |  | -0,0333 | 0,0459 | -0,0015 | 0,0108 |
| Unemployed | 0,0020 | -0,1689 | -0,0003 | 0,0041 |  | 0,0195 | -0,1701 | -0,0033 | 0,0235 |
| Retired | 0,0014 | -0,1941 | -0,0003 | 0,0032 |  | 0,0175 | -0,1359 | -0,0024 | 0,0168 |
| Other | -0,0388 | -0,0467 | 0,0018 | -0,0217 |  | 0,0042 | -0,0031 | 0,0000 | 0,0001 |
| Cádiz | -0,0055 | -0,1882 | 0,0010 | -0,0124 |  | -0,0013 | -0,2872 | 0,0004 | -0,0027 |
| Córdoba | 0,0167 | 0,0589 | 0,0010 | -0,0117 |  | -0,0204 | 0,1737 | -0,0035 | 0,0252 |
| Granada | 0,0199 | 0,0761 | 0,0015 | -0,0181 |  | -0,0238 | -0,0199 | 0,0005 | -0,0034 |
| Huelva | -0,0141 | 0,0000 | -0,0003 | 0,0041 |  | -0,0142 | 0,0233 | -0,0003 | 0,0024 |
| Jaén | 0,0194 | 0,0137 | 0,0003 | -0,0032 |  | -0,0098 | 0,2006 | -0,0020 | 0,0140 |
| Málaga | 0,0294 | -0,0702 | -0,0021 | 0,0247 |  | -0,0383 | -0,0257 | 0,0010 | -0,0070 |
| Sevilla | -0,0474 | 0,0846 | -0,0040 | 0,0480 |  | -0,1576 | 0,0942 | -0,0148 | 0,1053 |
| Explained |  |  | -0,0743 |  |  |  |  | -0,1165 |  |
| Unexplained |  |  | -0,0094 |  |  |  |  | -0,0245 |  |
| CI |  |  | -0,0836 |  |  |  |  | -0,1410 |  |
