# Supplementary material for: Withstanding austerity: Equity in health services utilisation in the first stage of the economic recession in Southern Spain
Source: PLoS One. 2018 Mar 30;13(3):e0195293. doi: 10.1371/journal.pone.0195293 (PMC5877882; doi:10.1371/journal.pone.0195293)
Supplement: S2 Table — (DOC) [file pone.0195293.s003.doc]

**S2 Table**

Change in elasticity and distribution effect by variable category.

|  | **GP** | |  | **Specialist** | |  | **Hospitalisation** | |  | **Emergency** | |
| --- | --- | --- | --- | --- | --- | --- | --- | --- | --- | --- | --- |
|  | Change elasticity | Change distribution |  | Change elasticity | Change distribution |  | Change elasticity | Change distribution |  | Change elasticity | Change distribution |
| Sex | 0,0003 | 0,0029 |  | 0,0030 | 0,0009 |  | 0,0000 | 0,0035 |  | 0,0022 | 0,0023 |
| Young | -0,0021 | -0,0004 |  | 0,0020 | -0,0027 |  | -0,0014 | -0,0083 |  | 0,0029 | -0,0084 |
| Old | 0,0101 | 0,0008 |  | -0,0075 | -0,0030 |  | -0,0049 | -0,0017 |  | 0,0084 | -0,0026 |
| Poor self rated health | 0,0161 | 0,0014 |  | 0,0296 | 0,0033 |  | 0,0125 | 0,0079 |  | 0,0043 | 0,0042 |
| Poor mental health | -0,0080 | 0,0011 |  | -0,0118 | 0,0019 |  | -0,0074 | 0,0007 |  | -0,0138 | 0,0012 |
| One chronic condition | -0,0003 | 0,0004 |  | 0,0007 | 0,0003 |  | 0,0009 | 0,0001 |  | -0,0013 | 0,0002 |
| Two-three chronic conditions | 0,0026 | 0,0053 |  | 0,0043 | 0,0043 |  | 0,0020 | -0,0011 |  | 0,0034 | 0,0022 |
| Four or more chronic conditions | -0,0034 | 0,0042 |  | 0,0043 | 0,0023 |  | -0,0008 | 0,0007 |  | 0,0041 | 0,0026 |
| Accident | -0,0013 | 0,0001 |  | 0,0042 | -0,0048 |  | -0,0003 | -0,0035 |  | 0,0009 | -0,0123 |
| Income | -0,0238 | 0,0014 |  | -0,0275 | -0,0005 |  | -0,0481 | 0,0024 |  | -0,0147 | 0,0020 |
| Up to 5 yr education | 0,0062 | -0,0004 |  | 0,0043 | 0,0020 |  | -0,0117 | 0,0010 |  | -0,0021 | 0,0006 |
| Up to 8 yr education | -0,0003 | 0,0007 |  | -0,0023 | -0,0040 |  | 0,0029 | -0,0016 |  | 0,0005 | -0,0007 |
| Secondary studies | 0,0013 | 0,0004 |  | -0,0097 | -0,0010 |  | 0,0132 | -0,0010 |  | -0,0036 | 0,0002 |
| University studies | 0,0039 | 0,0002 |  | -0,0237 | -0,0005 |  | 0,0114 | -0,0001 |  | 0,0037 | -0,0001 |
| Health insurance | 0,0026 | 0,0000 |  | -0,0059 | 0,0006 |  | -0,0026 | 0,0007 |  | -0,0026 | -0,0001 |
| Municipality pop. 10 to 50,000 | 0,0004 | -0,0005 |  | 0,0016 | -0,0001 |  | -0,0025 | 0,0006 |  | -0,0016 | -0,0007 |
| Municipality pop. > 50000 | 0,0001 | -0,0001 |  | -0,0007 | -0,0042 |  | 0,0037 | 0,0040 |  | 0,0016 | -0,0012 |
| Province capital | 0,0009 | -0,0007 |  | 0,0010 | -0,0001 |  | 0,0055 | -0,0040 |  | 0,0022 | -0,0029 |
| Unemployed | -0,0028 | 0,0000 |  | -0,0004 | 0,0000 |  | 0,0058 | 0,0046 |  | -0,0029 | 0,0000 |
| Retired | 0,0055 | 0,0019 |  | -0,0050 | 0,0074 |  | -0,0010 | 0,0059 |  | -0,0031 | 0,0010 |
| Other | -0,0001 | 0,0001 |  | -0,0006 | 0,0020 |  | 0,0016 | 0,0009 |  | -0,0020 | 0,0002 |
| Cádiz | -0,0039 | -0,0013 |  | 0,0029 | 0,0054 |  | 0,0128 | 0,0002 |  | -0,0008 | 0,0001 |
| Córdoba | 0,0008 | 0,0046 |  | 0,0050 | 0,0033 |  | 0,0017 | 0,0014 |  | -0,0022 | -0,0023 |
| Granada | -0,0031 | 0,0014 |  | 0,0047 | -0,0007 |  | -0,0010 | 0,0021 |  | -0,0033 | 0,0023 |
| Huelva | -0,0003 | 0,0002 |  | 0,0007 | 0,0001 |  | -0,0011 | -0,0003 |  | 0,0000 | -0,0003 |
| Jaén | 0,0001 | 0,0047 |  | 0,0009 | 0,0056 |  | -0,0009 | -0,0031 |  | -0,0004 | -0,0018 |
| Málaga | 0,0053 | 0,0012 |  | -0,0091 | 0,0022 |  | 0,0095 | -0,0018 |  | 0,0047 | -0,0017 |
| Sevilla | -0,0061 | -0,0031 |  | 0,0051 | 0,0000 |  | -0,0076 | -0,0006 |  | -0,0093 | -0,0015 |
